# TRANSPORTING CHILDREN IN CARS AND THE USE OF CHILD SAFETY RESTRAINT SYSTEMS

**DOI:** 10.1590/1413-785220162405140570

**Published:** 2016

**Authors:** ALLAN QUADROS GARCÊS, IGOR BONIFACIO ANDRADE COIMBRA, DIEGO SALVADOR MUNIZ DA SILVA

**Affiliations:** 1. Universidade Federal do Maranhão, São Luís, MA, Brazil.

**Keywords:** Accidents, traffic. Child. Child restraint systems. Seat belts. Accident prevention.

## Abstract

**Objective:**

To evaluate the transport of children in automobiles and the use of child restraints systems (CRS).

**Methods:**

This is a transversal descriptive study which included 200 vehicle drivers who carried 0-10 year old children in the city of São Luis, MA, Brazil. The drivers' passengers' and children's features were properly identified. The children's transportation using CRS were analyzed according to the Resolution 277/8 of the Brazilian National Traffic Department.

**Results:**

The transportation of children was classified as inappropriate in 70.5% of the vehicles analyzed. The most common way for children transportation was free on the back seats (47%) or on the lap of passengers/drivers (17%). The main reasons to justify the improper transportation were either not understanding the importance of CRS use (64.5%) or not having financial resources to buy the devices. The child safety seat was the most used CRS (50.8 %) among vehicles with proper child transportation system.

**Conclusion:**

The transportation of children was inappropriate in most of the vehicles analyzed, reflecting the need for creating awareness among automobile drivers, including education, supervision and improvement of policies for health improvement and prevention of accidents involving children transportation. Level of Evidence III, Cross Sectional Study.

## INTRODUCTION

Trauma is the leading cause of death and disability in pediatric patients, and it is considered an important public health problem in Brazil and in the world.^1^ ^,^ ^2^ Estimates indicate a growing trend in mortality due to road accidents in the world. It is expected that by 2030 the indicators increase by 40% if effective preventive measures are not taken.^3^ These alarming figures are due to the progressive increase of the number of circulating vehicles, growth of urban populations, lack of popular culture focused on safety, impunity, lack of effective legislation and poor condition of circulation roads.^4^

Several morphological, functional and biological characteristics inherent to childhood predispose children to car accidents as decisive factors for the discernment of the traffic conditions are still under development, and the smaller stature of children hinders the perception of their presence by the drivers.^5^

To minimize deaths and sequelae among children as car passengers, child restraint systems (CRS) or child safety seats were developed, popularly known as infant car seat, toddler car seat or simply children car seat, among others.^6^ When properly used, CRS reduce mortality by 71%; however, the risk of serious injury doubles when using the wrong model of CRS.^7^

The use of safety seats reduces by 82% the occurrence of serious injuries and by 80% the risk of hospitalization due to car accidents.^8^ ^,^ ^9^ The restriction provided by CRS improves distribution of impact forces during collisions through the transmission to the child's most resistant body parts (shoulders and chest), control of trunk and skull excursion and preventing shock against the vehicle's people and parts or being thrown out of it. The device restricts inadequate spontaneous movement, inadvertent door opening by the child, exposure of body parts through the windows, intrusion by the child in the pilot area and reduces the child's position changes in fast decelerations and curves.^7^

The recognition of the importance of CRS by the National Traffic Council (*Conselho Nacional de Trânsito,* Contran) occurred through Resolution 277/08,^10^ which regulated the use of child restraint systems in the country. Thus, the mandatory use of CRS has been established through criteria based on the child's age and weight (in compliance with international standards).

The regulation of CRS is currently in force, however there is no information on adherence and the correct use of devices. Due to the relevance of this topic, this study aims to evaluate the transportation of children in vehicles and the use of CRS in the city of São Luis, MA, Brazil.

## METHODS

### Study design and sampling

This is a descriptive cross-sectional study performed with a convenience sample (non-probabilistic) formed by 200 drivers of automotive vehicles carrying children from birth to 10 years old in the city of São Luis, MA, Brazil.

Data collection was carried out in the avenues located near public and private schools during the month of August, 2014. A team of academic researchers of a Medical School was previously selected and trained. Through direct interviews, the vehicle driver was informed about the research, read and signed the Informed Consent Form. Then, thy were asked to respond the "Assessment Protocol of Children Transport in Vehicles" tool, developed by the researchers. Personal identification data were codified and kept confidential. Drivers who refused to participate in the study or did not agree to sign the Informed Consent were not included.

This study is in accordance with Resolution 466/12 of the National Health Council (*Conselho Nacional de Saúde*) and was approved by the Ethics Committee of *Universidade Federal do Maranhão*, under number 698,695.

### Variables analyzed

The variables regarding the vehicle drivers and passengers were: drivers' age and gender, safety belt use and number of passengers in the vehicle. The degree of relatedness of the driver and the child was stratified as father (biological, adoptive or step-father), mother (biological, adoptive or step-mother), uncle or aunt, grandfather or grandmother, and others (brother, taxi driver, etc.). The drivers level of education was categorized as illiterate, basic schooling (elementary school, complete or incomplete), medium schooling (high school, complete or incomplete) and higher education (college or university education, complete or incomplete).

The children's age, gender, weight and height were informed by the vehicle driver. The number of children in the vehicle and the transportation mode were evaluated according to the Contran Resolution 277/08.^10^ This regulation states that children up to one year old should be transported in infant car seat devices in the back seat facing the rear window, with a slight slope. Children aged between one and four years old must use toddlers' safety car seat. Children between four and seven years old must use booster seats fixated in the back seat with three-point safety seat belts. Children over seven years old should use seat belts. Children aged 10 years or older are allowed to travel in the front seat.^10^ According to this regulation, child's transportation was classified as appropriate or inappropriate in each case.

When children transportation was considered inappropriate, irregularities were classified as: child in the lap, loose (sitting in the car seat without any CRS), using seat belt (inappropriate use according to the child's age, weight or height) or in a child's car seat (inappropriate use according to the child's age, weight or height) or standing (children standing between the driver and passenger seats). In cases of inadequacy, the vehicle's driver was questioned about the absence of CRS.

### Data analysis

Statistical analysis was performed using the software Epi-Info 7.1.3. The averages, absolute and relative frequencies and standard deviation (SD) are presented in tables and figures. Microsoft Word and Excel software were also used for editing text and tables.

## RESULTS

Table 1 shows the drivers' and passengers' characteristics of the 200 vehicles transporting children that have been approached. Most drivers were male (79.5%), and were the child's father (71%), aged between 31 and 40 years old (39%), with mid level education (58%) and were using safety belts (74%). Besides drivers and children, vehicles were carrying two other passengers (46%), who also used seat belts (52.5%).

**Table 1:** Sample characterization of drivers and passengers of vehicles transporting children.

Variables	n	%
Drivers' gender		
Male	159	79.5
Female	41	20.5
Degree of relativeness of the driver to the child		
Father	142	71
Mother	26	13
Uncle or aunt	15	7.5
Grandparents	11	5.5
Other	6	3
Drivers' age (years old)		
18-30	69	34.5
31-40	78	39
41-50	40	20
51-60	8	4
Above 60	5	2.5
Mean ± standard deviation	35.86 (±9.34)	
Drivers' schooling		
Illiterate	1	0.5
Basic education	2	1
Medium education	116	58
Higher education	81	40.5
Driver used seat belt		
Sim	148	74
Não	52	26
Uso de cinto pelo passageiro		
Yes	105	52.5
No	95	47.5
Passenger used seat belt		
1	29	14.5
2	92	46
3	51	25.5
4 or more	28	17
Mean ± Standard deviation	2.43 (±1.02)	

Acta Ortop Bras. 2016;24(5):275-8

The 200 vehicles' drivers were carrying 293 children, corresponding to 1.46 ± 0.93 children per vehicle. Most vehicles (71.5%) were carrying only one child (71.5%) of the male gender (51.9%). Most children were aged between one and four years (47.4%) (Table 2). The children's weight and height was 15.76 ± 8.11 kg and 87.60 ± 28.30 cm, respectively.

**Table 2:** Sample characterization of children transported in vehicles.

Variables	n	%
Number of children		
1	143	71.5
2	36	18
3	12	6
4 or more	9	4.5
Mean ± Standard deviation	1.46 (±0.93)	
Gender		
Male	152	51.9
Female	141	48.1
Age (years old)		
Up to 1	55	18.8
>1 and ≤4	139	47.4
>4 and ≤7	78	26.6
>7 and ≤10	21	7.2
Mean ± Standard deviation	3.65(±2.24)	

**Figure 1:**
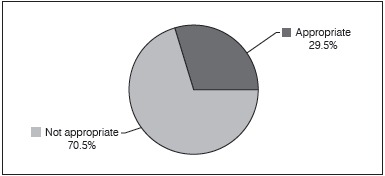
Analysis of children transportation in vehicles.

Child transportation was inappropriate in 70.5% of the vehicles approached (Figure 1). The main given reasons were "not finding important to use CRS" (64.5%) and "not have financial resources for purchasing CRS" (14.9%). Seven drivers (5%) declared they did not know about CRS or their importance. (Table 3)

**Table 3:** Justifications for inadequacy of children's transportation.

Justifications for inadequacy	n	%
Does not consider it as important	91	64.5
Has no financial resources to buy a CRS	21	14.9
The vehicle has only two seats	9	6.4
Does not know about CRS	7	5
Child did not adapt/has accepted CRS	5	3.5
Will provide/buy a CRS	3	2.1
Owns a CRS, but does not use it due to lack of inspection	3	2.1
CRS occupy too much space inside the vehicle	2	1.4

Most children were transported in the vehicle's back seat (88.4%) and in the central seat (35.2%). The most common children transportation way was loose in the back seat without any restraint equipment (safety belt or CRS) (47%) or in the passenger's or driver's lap (17.5%). Thirty-two children (10.9%) were transported on the front passenger seat and two (0.7%) in the driver's lap, while driving the vehicle.

The assessment of the current situation found that in 46.5% of cases, the correct way to transport children would be a toddler safety seat and 25.5% in a booster car seat with a seat belt. (Table 4)

**Table 4:** Comparison between the current and appropriate form to transport children in vehicles.

Transportation form	Current		Appropriate	
	n	%	n	%
Loose	94	47	-	-
On the lap	34	17	-	-
Infant safety seat	31	15.5	46	23
Toddler safety seat	27	13.5	93	46.5
Booster seat	5	2.5	51	25.5
Vehicle´s seat belt	8	4	9	4.5
Other	1	0.5	1	0.5

Of the 59 vehicles considered adequate for transporting children, the infant safety seat (50.8%) and the toddler safety seat (44.1%) were the most used CRS.

## DISCUSSION

In this study, children transportation was inappropriate in most vehicles that have been approached, similar to the findings of other studies.^1^ ^,^ ^6^ ^,^ ^11^ ^-^ ^13^ The lack of CRS or inadequate ones can lead to serious injuries or death of children in cases of collisions, since the child is more fragile and lacks defensive attitudes or danger perception.^5^ ^,^ ^14^

Traffic accidents are one of the most important factors influencing the morbidity and mortality of children in the country. A study conducted in a referral trauma center in Embu and Taboão da Serra (SP, Brazil), from December 2005 to December 2006 showed that 15% of the trauma mechanisms in childhood were related to traffic accidents.^15^ In San Diego (USA), according to information obtained from the database of the Legal Medical Service between January 2000 and December 2006, car accident was the leading cause of death (40.2%) in children and adolescents, followed by asphyxia and penetrating trauma.^16^

In a retrospective study conducted in Uberlândia (MG, Brazil), 1,123 victims of traffic accidents under the age of 15 were treated at *Hospital de Clínicas* from 1999 to 2003. It was found that 58.8% were not using safety devices and/or used them incorrectly at the time of the accident.^4^ These findings reinforce the need for the CRS and seat belts use for children and adolescents as one measure to reduce morbidity and mortality associated to traffic accidents.

Oliveira et al.^6^ found that 42.7% of children enrolled in kindergartens of Maringá (PR, Brazil) were inappropriately transported with CRS. The errors were the presence of two or more children in the vehicle (odds ratio = 5.10, p = 0.007), lower parental education level and income (medium average income and education: odds ratio = 7.00, p = 0.003; lower average income and education: odds ratio = 3.40, p = 0.03).

In this study, most vehicle drivers were the child's father, well-educated and were wearing the seat belt. However, 26% of drivers and 47.5% of passengers were not using seat belts. The lack of seat belt use by the parents can contribute to the non-use by children and adolescents,^7^ ^,^ ^14^ ^,^ ^17^ pointing out the need for educational and preventive policies showing the importance of safety equipment for all vehicle occupants.^18^ ^,^ ^19^

Most children were loose in the back seat without any containment equipment or CRS, but 32 children were in the front passenger seat and two in the driver's lap, despite the mandatory use of CRS and seat belt in the back seat, stated by Contran Resolution 277/08. Transporting children in the front passenger seat is permitted only in special situations (vehicles without rear seat, for example), while using appropriate CRS.^10^ The transport of children in the driver's lap is inadmissible, due to the high life threatening risk of the vehicle occupants and pedestrians. It is worth mentioning that the Contran Resolution has serious limitations for not considering the child's weight in the correct CRS use and for not encouraging the placement of infants facing the vehicle's rear window for a longer time.^6^

In vehicles suitable for transporting children, the most common retaining device was the infant safety seat, suitable for children aged up to one year old,^10^ while in the city of Maringa,^11^ the toddler's safety car seat was the most used CRS. Differences between the two studies are explained by the methodological and characteristics differences of two samples.

Most drivers do not consider important to use CRS, showing a lack of appreciation and understanding by the population of the importance of such device. The use of safety belt and CRS have a major impact on hospital costs and rehabilitation.^19^ Therefore, to raise awareness on the proper use of seat belts and CRS among the population should be a commitment of all health professionals in order to reduce the number of child victims of car accidents.

The present study has some limitations, such as lack of a sample size calculation, the small number of participants and the non-probabilistic sampling model that hinders the generalization of the results. However, due to the lack of national data on the subject it becomes relevant to reveal aspects of children automobile transport in the city of São Luis, MA, Brazil, a city in northeastern Brazil, which may reflect the situation in other parts of the region. These data are even more important when one realizes the relevant precariousness in health care.

This study may help to create awareness among drivers and health professionals and it serves as a quantitative data showing the seriousness of the problem, and may be useful to support educational programs on safe transportation of children, especially in the family and school context. The results also highlight the need to improve the inspection regarding CRS, enforcing the existing legislation on effective security measures for children transportation among the population.

Proper child transportation with the correct use of CRS establishes safety conditions that can dramatically reduce the chances of severe traumatic injury and death in the event of collisions.

## CONCLUSION

The transportation of children was inadequate in 70.5% of the vehicles approached. It is necessary to create awareness among drivers, to increase inspection and improve public policies for health promotion and prevention of traffic accidents.
